# The effect of dapagliflozin ointment on induced psoriasis in an experimental model

**DOI:** 10.25122/jml-2023-0084

**Published:** 2024-03

**Authors:** Waleed Khaled Younis Al bahadly, Ahlem Bdioui, Moaed Al-Gazally, Haider Al-Saedi, Sihem Hmissa Belhaj Salah, Mukhallad Ramadhan

**Affiliations:** 1 Department of Pharmacology, College of Pharmacy, University of Al-Ameed, Karbala, Iraq; 2 Department of Physiology, Faculty of Medicine Ibn Al Jazzar, University of Sousse, Sousse, Tunisia; 3 Department of Clinical Biochemistry, College of Medicine, University of Al-Ameed, Karbala, Iraq; 4 Department of Pathology, College of Medicine, University of Misan, Misan, Iraq

**Keywords:** imiquimod, dapagliflozin, psoriasis, mice

## Abstract

Dapagliflozin is a pharmacological drug commonly used to manage type 2 diabetes by inhibiting the sodium-glucose cotransporter in the proximal renal tubules. The primary objective of this research was to develop a topical ointment formulation containing dapagliflozin and assess its efficacy in treating psoriasis using an imiquimod-induced psoriasis model. A total of 16 Swiss albino mice, with weights ranging from 24 to 30 grams, were allocated randomly into six groups, each group including ten animals. The study assessed the effects of various concentrations of dapagliflozin ointment on levels of tumor necrosis factor-alpha (TNF-alpha), interleukin-8 (IL-8), IL-17, and IL-37, as well as on erythema, scaling, and epidermal thickness. Dapagliflozin ointment significantly reduced these cytokine levels and disease scores, indicating anti-psoriatic and anti-inflammatory properties. Therefore, when applied topically, dapagliflozin ointment had strong efficacy against imiquimod-induced psoriatic skin inflammation, suggesting its potential as a novel therapeutic option for psoriasis treatment.

## INTRODUCTION

Psoriasis is a chronic, immune-mediated inflammatory skin disorder characterized by the presence of erythematous plaques primarily located on the extensor surfaces. These plaques are frequently accompanied by pruritus and are covered with white scales [1]. The prevalence of a common chronic skin condition is subject to considerable variation based on environmental factors, patient age, and gender. Its prevalence ranges from 0.91% to 8.5% in adults, and in children, it ranges from 0.1% to 2.1%. The incidence rate among adults is about 40.8 per 100,000, ranging from 78.9 to 230 per 100,000 in children [2]. The importance of cytokines in the development of psoriasis has led to the development of targeted interleukin modulators that seek to restore the appropriate growth and activity of keratinocytes, thereby influencing the management of psoriasis [3]. Sodium-glucose cotransporter 2 (SGLT-2) inhibitors, primarily used as oral glucose-lowering agents in type 2 diabetes, have shown benefits in weight reduction, blood pressure control, and improved insulin resistance and glucose tolerance [4]. Dapagliflozin inhibits SGLT-2, an enzyme that reduces glomerular-filtered glucose and salt reabsorption, used to treat hyperglycemia in type 2 diabetes patients [5,6]. Koch *et al*. [7] emphasized the importance of dapagliflozin as an antioxidant and anti-inflammatory drug. The potential for dapagliflozin to modulate cytokine inflammatory pathways in psoriasis remains an area of exploration. Consequently, we aimed to assess the impact of topical dapagliflozin application on experimentally induced psoriasis. This assessment involved measuring the potential anti-psoriatic activity and the anti-inflammatory effects of this compound and conducting physical and histological evaluations.

## MATERIAL AND METHODS

### Preparation of dapagliflozin ointment

Dapagliflozin was prepared as an ointment by dissolving it in a mixture of concentrated ethanol, glycerol, and vaseline. This mixture was mixed at 70°C, cooled, and stirred for 30 minutes until solidified. The ointment was filtered, and sensory properties, pH, thermal, and centrifugal stability tests were performed. Rheological and microbiological tests were conducted according to the procedure of Jahandideh *et al*. [8].

### Experimental design

Sixty Swiss albino mice, weighing between 24 to 30 grams, were randomly divided into six groups (*n* = 10 mice in each group), and the dorsal region of each mouse was shaved over a 2 cm area.

**Control group (group 1):** received daily topical application of 0.5 mg vaseline ointment for 6 days on the shaved area.

**Induction group (imiquimod group 2):** 62.5 mg of 5% imiquimod cream was applied daily for 6 days to induce psoriasis.

**Clobetasol standard group (group 3):** 62.5 mg of 5% imiquimod cream was applied daily with 0.05% clobetasol propionate ointment daily for 6 days.

**Dapagliflozin (5%) treatment group (group 4):** 62.5 mg of 5% imiquimod cream was applied daily with dapagliflozin ointment at concentrations of 5% and applied daily for 6 days.

**Dapagliflozin (10 %) treatment group (group 5):** 62.5 mg of 5% imiquimod cream was applied daily with dapagliflozin ointment at concentrations of 10% and applied daily for 6 days.

**Dapagliflozin (20 %) treatment group (group 6):** 62.5 mg of 5% imiquimod cream was applied daily with dapagliflozin ointment at concentrations of 20% and applied daily for 6 days.

These groups were euthanized on the seventh day. The alterations in skin appearance, specifically erythema and desquamation, were documented over six consecutive days. On the seventh day of the experiment, mice were anesthetized using ketamine (0.08 ml) and xylazine (0.16 ml), and blood samples were collected via cardiac puncture. Blood samples were centrifuged at 5,000 rpm for 10 minutes for serum separation. The levels of cytokines-tumor necrosis factor-alpha (TNF-alpha), interleukin-8 (IL-8), interleukin-17 (IL-17), and interleukin-37 (IL-37) -were measured using ELISA kits from Sunlong. Additionally, skin samples were obtained from the treated area. The skin samples were stored in a 10% formalin solution (Fluka) and sectioned and stained with hematoxylin and eosin (H&E) to examine histological alterations. Following this, the samples were subjected to cold centrifugation at 5,000 rpm for 10 minutes. The serum samples were collected and stored at a temperature of -80°C. This was done to facilitate the measurement of various biomarkers, specifically TNF-alpha, IL-8, IL-17, and IL-37.

### Clinical symptoms follow-up

**Figure 1:**
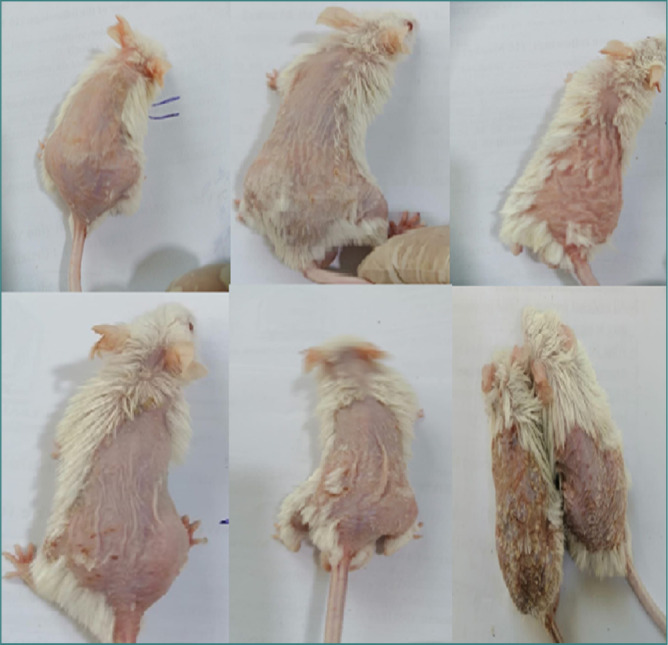
The shaved back of mice

The severity of psoriasis symptoms in mice was monitored daily. This included assessing the redness and scaling intensity on the skin using a standardized scale (Figure 1). The values ranged from 0 to 4, where 0 indicates no symptoms, 3 indicates very noticeable symptoms, and 4 indicates very severe symptoms. A light microscope was used to evaluate the histopathological texture of the skin and quantify the thickness of the histological cortical layer.

### Statistical analysis

SPSS 22 was employed for data analysis. Descriptive statistics included calculating the mean and the standard error of the mean (SEM) for numerical data. To evaluate numerical data, a one-way analysis of variance (ANOVA), or an independent t-test was used. *P* values below 0.05 were considered statistically significant.

## RESULTS

The color, liquefaction, viscosity, and skin irritation properties of the dapagliflozin compound ointment were evaluated. The findings demonstrated that dapagliflozin at 5%, 10%, and 20% concentrations did not liquefy, alter color, or phase separate after three months of ointment formation. Skin irritation tests indicated no redness or irritation when applied in varying amounts.

### Severity of psoriasis score

**Figure 2:**
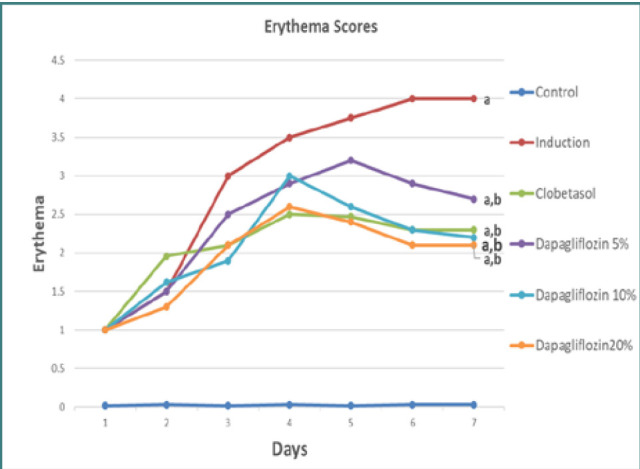
Comparative analysis of erythema scores across groups

**Figure 3:**
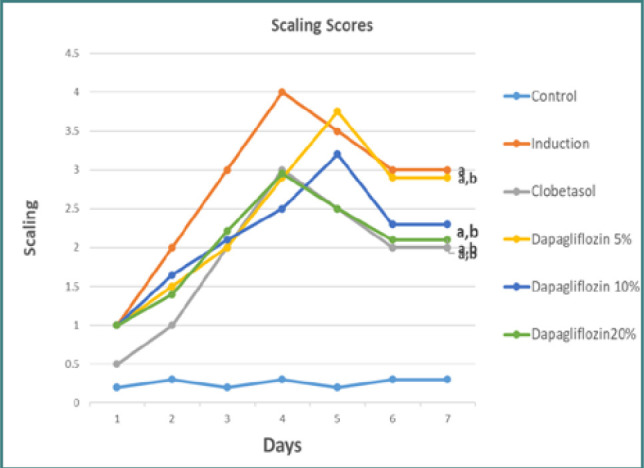
Comparative analysis of scaling scores across study groups

The experimental group that underwent psoriasis induction had a significant increase in erythema and crusting severity compared to the control group, as seen in Figure 2. Clobetasol treatment resulted in a significant reduction in the severity of psoriasis symptoms, including erythema and crusting. In the groups treated with dapagliflozin cream at concentrations of 5%, 10%, and 20%, a significant improvement in visual abnormalities was observed over the course of the treatment (*P* < 0.05). According to Figure 2, when comparing the efficacy of dapagliflozin ointment formulations (5%, 10%, and 20%) and clobetasol ointment (0.05%), no statistically significant differences were found in the severity of psoriatic lesions (figure 3).

### Serum biomarkers

As demonstrated in Table 1, 5% imiquimod significantly increased (*P* <0.05) the concentration of serum cytokines (TNF-alpha, IL-8, IL-17, and IL-37) compared to the control group. Treatment with clobetasol ointment significantly decreased these cytokine levels compared to the imiquimod group (*P* ≤ 0.05). The serum levels of all these biomarkers were significantly lower in the group that developed psoriasis (*P* ≤ 0.05), with a 0.5% difference between dapagliflozin ointment and clobetasol ointment. The three ointment concentrations (5%, 10%, and 20%) restored certain cytokines to their normal levels compared to the control group.

**Table 1:** The effect of vaseline, imiquimod, clobetasol and dapagliflozin (5%,10%,20%) ointment on inflammatory biomarkers Skin histological changes

Groups	TNF-alpha	IL-8	IL-17	IL-37
Control	48.50 ± 1.83	22.65 ± 0.78	31.92 ± 1.74	29.63 ± 0.98
Imiquimod (IQ)	269.38 ± 6.38^a^	86.35 ± 2.76^a^	113.46 ± 3.76^a^	74.03 ± 2.12^a^
Clobetasol (CL)	142.80 ± 3.26^ab^	57.56 ± 1.82^ab^	69.19 ± 2.67^ab^	53.45 ± 1.48^ab^
Dapagliflozin (DP, 5%)	90.95 ± 4.72^abc^	34.84 ± 1.56^abc^	41.35 ± 3.28^abc^	45.43 ± 1.78^ab^
Dapagliflozin (DP, 10%)	90.95 ± 4.72^abc^	34.84 ± 1.56^abc^	41.35 ± 3.28^abc^	60.43 ± 1.78^ab^
Dapagliflozin (DP, 20%)	89.03 ± 5.88^abc^	36.74 ± 2.52^abc^	51.04 ± 3.32^abc^	48.88 ± 1.57^ab^

Data are presented as mean ± standard deviation (SD). The superscript letters indicate statistical significance: 'a' denotes P ≤ 0.05 compared to the control group; 'b' denotes P ≤ 0.05 compared to the imiquimod (IQ) group; 'c' denotes P ≤ 0.05 compared to the clobetasol (CL) group

### Skin histological changes

**Figure 4:**
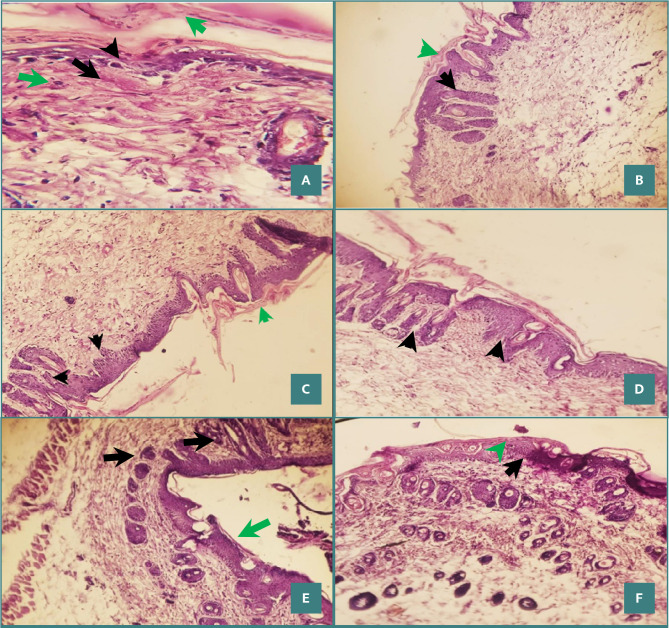
Histopathological effects of dapagliflozin against imiquimod across study groups. A, Control group with normal histology. B, Imiquimod (5%) treated group showing pronounced histological changes with Munro microabscesses (black arrows) and epidermal proliferation (green arrow). C, Clobetasol (0.05%) ointment group with diminished epidermal thickening. D and E, Dapagliflozin (5% and 10%, respectively) ointment groups with unaltered keratin and epidermal layers. F, Dapagliflozin (20%) group with significant histological improvement compared to the imiquimod group.

The histological features in the control group included keratin layer growth without Munro's inflammation and a lack of alterations in epidermal layer structure thickness. Imiquimod (5%) caused severe histological alterations indicated by Munro's inflammation with epidermal layer expansion toward the dermis (P <0.05) when compared to the control group. The histological characteristics of the clobetasol ointment group (0.05%) resulted in a weakening of the epidermal thickness and a reduction in inflammatory symptoms. Furthermore, the dapagliflozin ointment groups (5% and 10%) had normal keratin growth and epidermal thickness survival, whereas these indicators were decreased in the imiquimod group (Figure 4 A–F). Compared to the imiquimod group, the dapagliflozin ointment group had a clear effect (20%) on correcting epidermal form, thickness, and keratin layer.

## DISCUSSION

In this study, we observed that mice treated with imiquimod developed inflammation, erythema, and scaling, a reaction consistent with the effects of imiquimod as documented in the literature [9]. Imiquimod treatment increased skin thickness and triggered alterations predominantly through the activation of key pro-inflammatory cytokines such as TNF-alpha and IL-17, which are known to induce further cytokines like IL-6 and IL-8 [10,11]. IL-8 is associated with inflammation and angiogenesis, while IL-37 may promote immune cell activation and exacerbate inflammatory disorders [12,13]. Furthermore, this study demonstrated that clobetasol effectively reduced erythema and scaling, likely due to its vasoconstrictive properties, which reduce erythema by suppressing histamine and bradykinin receptors [14]. Furthermore, the observed effects included a decrease in the proliferation of keratinocytes and growth factors, which may be attributed to the inhibition of fibroblast proliferation, migration, chemotaxis, and protein synthesis. Additionally, signs of dermal atrophy were evident [15,16]. The selection of different concentrations of dapagliflozin ointment was based on prior pilot research conducted to determine the concentrations included in the present study. Dapagliflozin has anti-inflammatory effects by reducing plasma cytokine levels, irrespective of diabetes mellitus [17]. Dapagliflozin has been shown to have additional anti-inflammatory and antioxidant stress properties [18].

The present study demonstrated that the administration of dapagliflozin resulted in a decrease in scaling and erythema ratings in the imiquimod-induced psoriasis model. The therapeutic benefits of topical dapagliflozin may be attributed to its possible vasoconstrictive influence, reducing erythema [19]. Dapagliflozin had a mitigating effect on the scaling induced by imiquimod. This outcome might be attributed to the induction of cell cycle arrest and a subsequent decrease in cellular proliferation. The effects of dapagliflozin contributed to its potential ability to decrease levels of TNF-α, IL-8, IL-17, and IL-37 [20]. Histological analyses confirmed the substantial improvement and suggested a dose-response relationship, with higher dapagliflozin concentrations showing increased antipsoriatic activity.

## CONCLUSION

When applied topically, dapagliflozin ointment displayed strong anti-psoriatic and anti-inflammatory effects in mice with imiquimod-induced psoriasiform skin inflammation.

## Personal thanks

The authors thank the University of Al-Ameed for the partial support.
